# No relationship between thymidine phosphorylase (TP, PD-ECGF) expression and hypoxia in carcinoma of the cervix

**DOI:** 10.1038/sj.bjc.6602882

**Published:** 2005-11-29

**Authors:** P Kabuubi, J A Loncaster, S E Davidson, R D Hunter, C Kobylecki, I J Stratford, C M L West

**Affiliations:** 1 Academic Department of Radiation Oncology, The University of Manchester, Christie Hospital NHS Trust, Manchester M20 4BX, UK; 2 Department of Clinical Oncology, Christie Hospital NHS Trust, Manchester M20 4BX, UK; 3 Experimental Oncology Group, School of Pharmacy and Pharmaceutical Sciences, The University of Manchester, Oxford Road, Manchester M13 9PL, UK

**Keywords:** thymidine phosphorylase, hypoxia, hypoxia-inducible factor, cervix

## Abstract

The expression of hypoxia-regulated genes promotes an aggressive tumour phenotype and is associated with an adverse cancer treatment outcome. Thymidine phosphorylase (TP) levels increase under hypoxia, but the protein has not been studied in association with hypoxia in human tumours. An investigation was made, therefore, of the relationship of tumour TP with hypoxia, the expression of other hypoxia-associated markers and clinical outcome. This retrospective study was carried out in patients with locally advanced cervical carcinoma who underwent radiotherapy. Protein expression was evaluated with immunohistochemistry. Hypoxia was measured using microelectrodes and the level of pimonidazole binding. There was no relationship of TP expression with tumour pO_2_ (*r*=−0.091, *P*=0.59, *n*=87) or pimonidazole binding (*r*=0.13, *P*=0.45, *n*=38). There was no relationship between TP and HIF-1*α*, but there was a weak borderline significant relationship with HIF-2*α* expression. There were weak but significant correlations of TP with the expression of VEGF, CA IX and Glut-1. In 119 patients, the presence of TP expression predicted for disease-specific (*P*=0.032) and metastasis-free (*P*=0.050) survival. The results suggest that TP is not a surrogate marker of hypoxia, but is linked to the expression of hypoxia-associated genes and has weak prognostic power.

Thymidine phosphorylase (TP), previously called PD-ECGF, is a 110 kDa protein homodimer which was originally isolated from platelets showing endothelial mitogenic activity (Miyazono *et al*, 1987). It was subsequently identified in other tissues including placenta, macrophages, lung, liver, spleen and peripheral lymphocytes (Yoshimura *et al*, 1990). Levels of the protein are elevated in solid tumours (Yoshimura *et al*, 1990; Takebayashi *et al*, 1996, 1999), rheumatoid arthritis synovium (Asai *et al*, 1993) and psoriatic lesions (Creamer *et al*, 1997).

Little is known about the regulation of TP expression, although certain cytokines are implicated in increasing its activity, including interleukin-1, tumour necrosis factor-*α*, fibroblast growth factor-*β* and interferon-*α* (Tevaearai *et al*, 1992; Eda *et al*, 1993; de Bruin *et al*, 2004). *In vitro* studies have shown that TP expression is also induced by hypoxia. Griffiths *et al* (1997)found TP expression in MDA 231 cells increased six-fold following 16 h growth in 0.3% oxygen). More recently, Abbas *et al* (2004) showed TP expression increased two-fold under hypoxia in human endometrial stromal cells. Cobalt stimulation increases TP levels, suggesting that its expression might be regulated by hypoxia-enhancer elements, that is, hypoxia-inducible factor (HIF) (Griffiths *et al*, 1997). Indeed, Sivridis *et al* (2002b) showed that HIF-2*α* overexpression was linked to TP expression in human endometrial adenocarcinomas. Interestingly, in the TP-overexpressing cell line RT112-TP, TP augmented the hypoxic induction of HIF-1*α* (Brown *et al*, 2005).

Thymidine phosphorylase has angiogenic properties (Moghaddam *et al*, 1995) which are dependent on its enzymatic activity (Miyazono *et al*, 1987; Miyadera *et al*, 1995). A relationship between TP expression and microvessel density, a histological measure of angiogenesis, has been reported in several solid tumours such as breast (Toi *et al*, 1995a), ovarian (Reynolds *et al*, 1994) renal (Imazano *et al*, 1997), and gastric (Takebayashi *et al*, 1996). In experimental models, TP overexpression increased tumour cell invasion (Ueda *et al*, 2001) and tumorigenicity (Griffiths and Stratford 1998; Brown *et al*, 2005). In man, high tumour expression of TP generally correlates with a poor prognosis in various solid malignancies (Takebayashi *et al*, 1996; Ikeda *et al*, 1999) including cervix cancer (Fujimoto *et al*, 1999; Hata *et al*, 1999; Ueda *et al*, 1999). However, this finding is not universal, as one study in cervix cancer showed that high tumour TP positivity was associated with a good prognosis (Oka *et al*, 2003). Of interest, high stromal TP expression correlated with a favourable prognosis in patients with colorectal carcinoma not receiving 5-FU based chemotherapy, which led the authors to suggest that TP produced by tumour cells and stromal macrophages may exert different roles (Yasuno *et al*, 2005).

The relationship between TP and VEGF is also of interest. There is evidence that VEGF may act downstream of TP (Brown *et al*, 2000), and that hypoxia augments the effect of TP on VEGF (Brown *et al*, 2005). The two proteins coexpress in breast carcinomas (Toi *et al*, 1995b) and this coexpression was the most potent angiogenic phenotype in endometrial carcinoma (Sivridis *et al*, 2002a). In contrast, an inverse relationship between TP and VEGF expression was found in cervical carcinomas (Tokumo *et al*, 1998) and their angiogenic activity appears highest when expressed relatively alone in human colon cancers (Takahashi *et al*, 1996).

In view of the uncertainty over the role of TP, the aims of this study were to examine the relationship of tumour TP expression with hypoxia, the expression of hypoxia-associated proteins and prognosis in patients with carcinoma of the cervix.

## MATERIALS AND METHODS

### Patients

Two series of patients were studied. Local ethical approval was obtained and full prior written consent was gained from all the patients. The first series of patients comprised 119 patients with locally advanced carcinoma of the uterine cervix treated with radical radiotherapy at the Christie Hospital NHS Trust between 1987 and 1993. Median follow-up was 60 months (range 27–115 months). Random cervical punch biopsies were taken prior to radical radiotherapy. Treatment was given according to standard techniques and dosages of the Manchester School (West *et al*, 1993). On completion of treatment they were reviewed following a standard protocol (a minimum of three monthly for the first year, four monthly for the second and third years, and six monthly in the fourth and fifth years). The sites of any disease relapse were identified clinically, radiologically, and histologically where appropriate. The recurrences were then classified as being either local (i.e. within the radiotherapy field) or metastatic.

The second series comprised a more recently treated group of 87 patients with locally advanced carcinoma of the cervix seen between May 1996 and December 1999. Tumour oxygenation data were obtained using an Eppendorf pO_2_ histograph as described elsewhere (Cooper *et al*, 1999).

### Pimonidazole binding

The method used for administration of pimonidazole to patients (Hypoxyprobe-1; NPI Inc. Belmont, MA, USA), taking tumour biopsies and staining have been described elsewhere (Nordsmark *et al*, 2001). Patients were biopsied 12–20 h after receiving pimonidazole. A semiquantitative scoring system was used to quantify pimonidazole binding as described elsewhere (Hutchison *et al*, 2004).

### TP expression

All staining was carried out on pretreatment biopsies. Immunohistochemical detection of TP was carried out using an immunoperoxidase method, as described elsewhere (Shimaoka *et al*, 2000). Formalin-fixed samples were embedded in paraffin and cut into 4 *μ*m thick sections. These were then deparaffinized with xylene, dehydrated using 100% ethanol, and washed with methanol. Endogenous peroxidase was blocked by immersion in 0.3% H_2_O_2_ in methanol for 30 min at room temperature (RT). The blocked sections were then washed in methanol, running water and three 5 min washes in TBS (pH 7.6). Slides were dried and 10% normal rabbit serum (DAKO XO902) was applied for 10 min at RT. The sections were incubated overnight at 4^o^C with a 1 : 30 dilution of primary mouse monoclonal antibody (gift from Adrian Harris). After washing, the slides were incubated for 30 min at RT with 1 : 400 rabbit anti-mouse biotinylated secondary antibody (DAKO EO413). The sections were then washed and incubated for 30 min at RT with streptavidin-biotin complex/horseradish peroxidase (sABC/HRP) in TBS. Following a further wash, the slides were incubated for 5 min with 0.5 mg ml^−1^ diaminobenzidine (DAB) in 0.03% H_2_O_2_. The sections were washed in distilled water and counterstained with haematoxylin before dehydrating and mounting.

The extent and distribution of TP staining was evaluated under low magnification, without the observer's knowledge of outcome data or oxygen measurements. The level of expression in the tumour cells was evaluated using a semiquantitative scoring system: 0 for absence of immunostaining, 1 for light staining, 2 for moderate staining, and 3 for heavy staining. Any staining of the tumour stroma was ignored in this assessment. All scoring was performed in a double-blind manner by two independent investigators.

### MVD, VEGF, HIF, CA IX, Glut-1

The staining and scoring methods for MVD (Cooper *et al*, 1998), VEGF (Loncaster *et al*, 2000), HIF-1*α* (Hutchison *et al*, 2004), HIF-2*α* (Beasley *et al*, 2002), CA IX (Loncaster *et al*, 2001) and Glut-1 (Airley *et al*, 2001) have been described elsewhere Only tumour nuclear HIF-1*α* was scored as described elsewhere (Aebersold *et al*, 2001). All scoring was performed in a double-blind manner by two independent investigators.

### Statistical analysis

Correlations between measurements were examined using Spearman's rank correlation. The distribution of TP score in relation to patient and tumour characteristics was investigated using Fisher's exact test. Survival was analysed using the Kaplan–Meier method and prognostic factors were assessed using log-rank analysis. Univariate and bivariate analyses were made of overall, metastasis-free and recurrence-free survival. Patients were stratified by their TP expression score as well as other putative prognostic factors (stage, age, differentiation). A stepwise multivariate Cox regression analysis was performed to further test the independence of TP from other parameters.

## RESULTS

### TP expression

Positive TP expression was seen in 92% (188/206) of the tumours. The expression was generally homogeneous within epithelial cells, and was predominantly cytoplasmic, with some nuclear staining. There was some degree of stromal TP expression thought to be owing to macrophage infiltration, but this was small relative to the tumour cytoplasmic staining. Higher levels of TP expression were seen adjacent to areas of necrosis, but no pattern in relation to tumour vasculature was observed.

### Scoring reproducibility

Intra-observer variation in scoring TP expression was tested by re-scoring all the tumour sections. There was a significant correlation between the two scores (*r*=0.92, *P*<0.001). Inter-observer variation was tested by a second observer scoring all the same sections. Again, there was significant correlation between the scores (*r*=0.88, *P*=0.001). Assessment of batch to batch variation showed sections stained on multiple occasions scored identically.

### TP expression and tumour oxygen measurements

In a series of 87 tumours, a median of four oxygen electrode tracks (range 1–7) was made per tumour, resulting in a median of 128 oxygen measurements (range 32–192). The median pO_2_ level was 4 mmHg (range 0–45 mmHg), and the median HP5 (percentage of values less than 5 mmHg) was 52% (range 0–96%). There was no relationship between TP expression and the Eppendorf measurements of tumour oxygenation. The correlations with median pO_2_ and HP5 were *r*=−0.091, *P*=0.59 and *r*=−0.014, *P*=0.93, respectively. There was no correlation between TP expression and pimonidazole staining (*r*=0.13, *P*=0.45, *n*=38).

### Relationship with angiogenesis and hypoxia-inducible markers

There was no relationship between TP expression and MVD (*r*=−0.15, *P*=0.10, *n*=120). There was a weak positive relationship between TP and VEGF expression (*r*=0.36, *P*=0.001, *n*=89). Expressions of both proteins were independent of disease stage and there was no significant difference between the distributions of the scores with stage for the two proteins. There was no significant relationship between TP and HIF-1*α* expression (*r*=−0.062, *P*=0.68, *n*=46). There were weak but significant positive relationships between TP expression and CA-IX (*r*=0.22, *P*=0.015, *n*=122) and Glut-1 (*r*=0.31, *P*=0.002, *n*=96). HIF-2*α* showed a weak borderline significant relationship with TP expression (*r*=0.34, *P*=0.09, *n*=26).

### Correlation with outcome

Table 1 summarises the distribution of tumour TP expression in relation to patient and tumour characteristics in 119 patients. Using Fisher's exact test, TP expression was independent of tumour stage and differentiation. Higher levels of expression were seen in tumour from younger patients.

**Figure 1:**
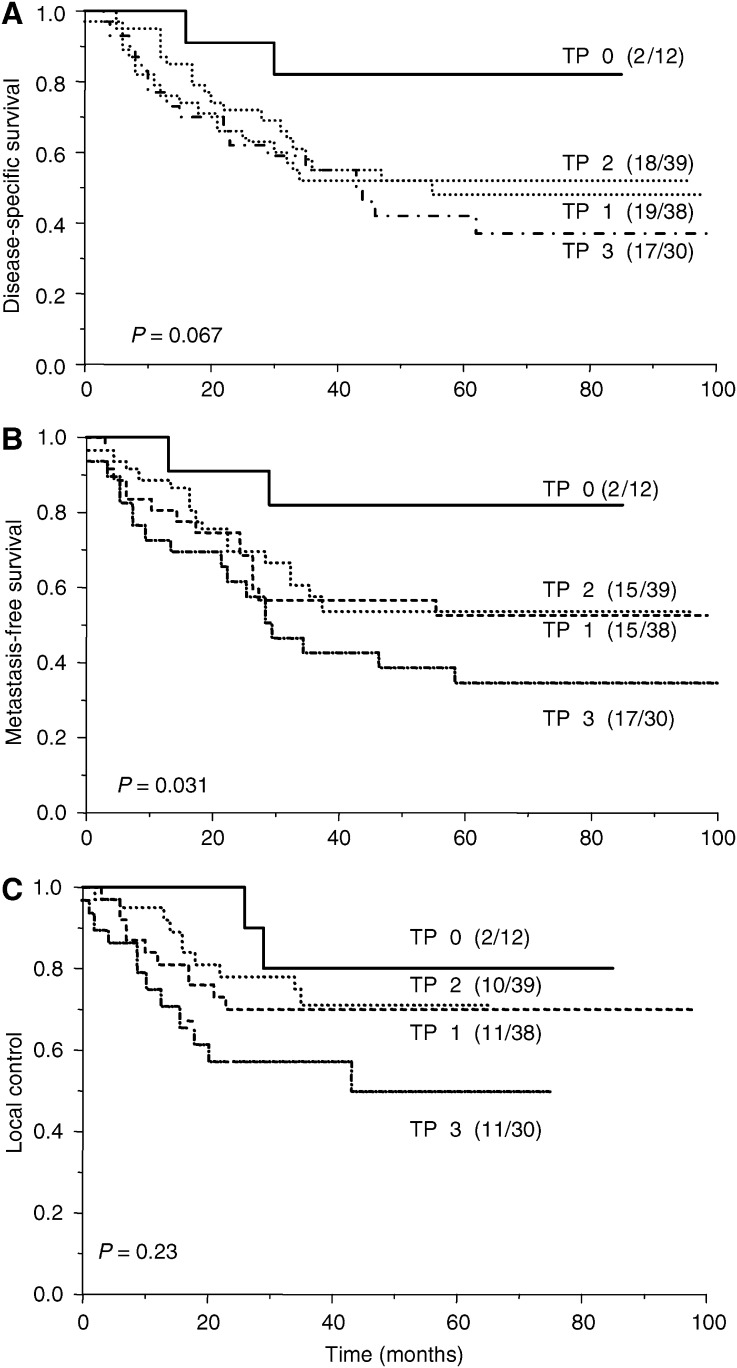
Disease-specific survival (**A**), metastasis-free survival (**B**) and local control (**C**), in relation to TP expression in 119 cervical cancer patients who received radical radiotherapy. Patients were stratified according to the intensity of TP expression (0=no staining, 1=light staining, 2=moderate staining and 3=heavy staining). The numbers of events and patients in each arm are indicated.

**Figure 2:**
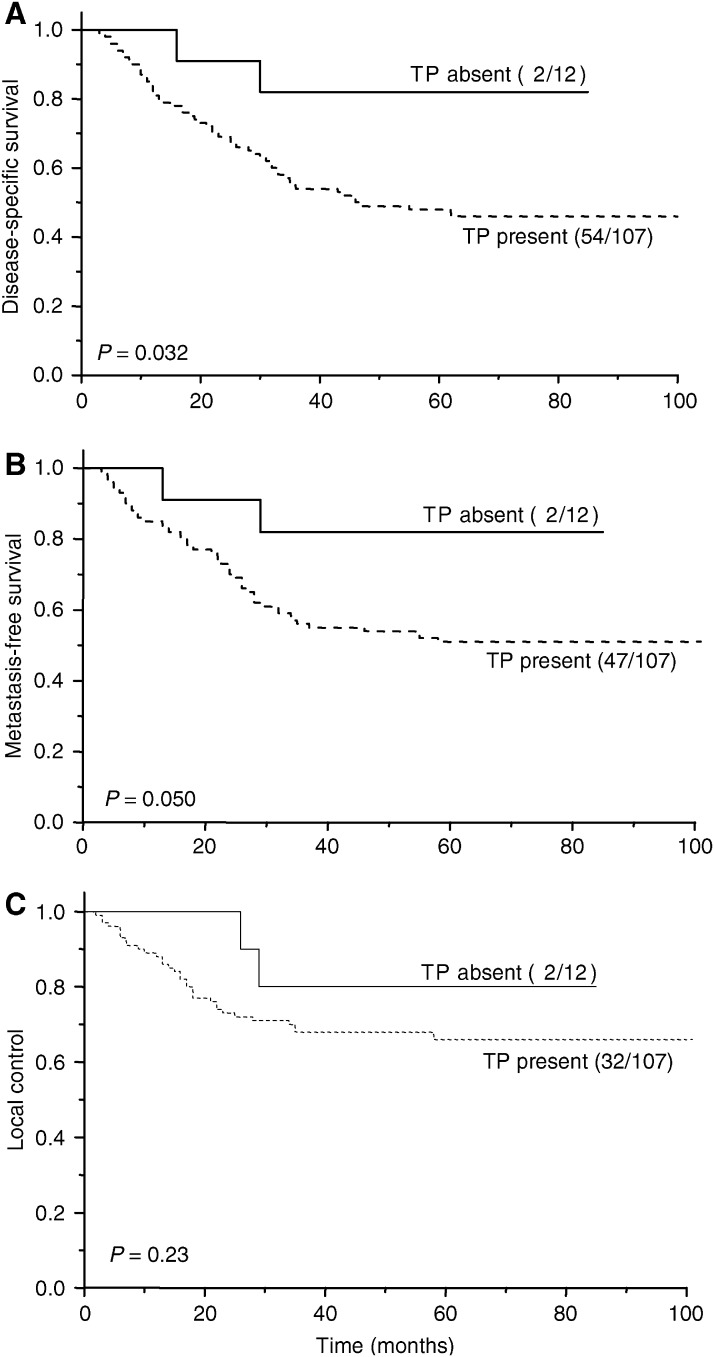
Disease-specific survival (**A**), metastasis-free survival (**B**) and local control (**C**) in relation to the presence or absence of TP expression in 119 cervical cancer patients who received radical radiotherapy. The numbers of events and patients in each arm are indicated.

Figure 1 illustrates the relationship between TP expression and treatment outcome. The level of expression was a significant prognostic factor for metastasis-free survival (*P*=0.031) and a borderline significant prognostic factor for disease-specific survival (*P*=0.067). There was no relationship with local control (*P*=0.23). The analyses were repeated stratifying patients by the presence or absence of immunostaining (Figure 2). The prognostic significance of TP expression was similar for both disease-specific (*P*=0.032) and metastasis-free (*P*=0.050) survival. The sensitivity of TP in predicting a poor outcome was 96% for both disease-specific and metastasis-free survival. However, the respective specificities were only 16 and 14%. The respective positive predictive values of tumour TP positivity were 50 and 44%. Table 2 summarises the results of univariate log-rank analyses of outcome following radiotherapy. Tumour stage was the strongest prognostic factor, followed by TP expression. Bivariate analyses were carried out examining the prognostic significance of TP expression after allowing for disease stage, tumour grade and patient age (Table 3). TP expression remained a weak predictor of disease-free and metastasis-free survival after allowing for disease stage, patient age and tumour grade. Adding the scores for TP and VEGF expression did not improve the prognostic ability over VEGF expression alone for disease-specific and metastasis-free survival (*P*=0.0057 and *P*=0.0024, respectively). However, the combined score was better at predicting local control than either VEGF or TP alone (*P*=0.029).

## DISCUSSION

The immunohistochemical detection of the intensity of TP expression has been shown to correlate with TP levels as determined by immunoassay (Fujimoto *et al*, 1999). The scoring method used in this study, based on the intensity of TP expression, was reproducible within and between scorers. The cytoplasmic distribution of TP in our study is consistent with series in uterine cancers (Fujimoto *et al*, 1999) and other malignancies (Reynolds *et al*, 1994; Moghaddam *et al*, 1995; Toi *et al*, 1995a; Takebayashi *et al*, 1996). However, some groups have described stromal macrophage TP expression in colorectal cancer (Saito *et al*, 2000) and adenocarcinoma of the lung (Kojima *et al*, 2002).

This is the only study, to the authors' knowledge, that has examined the relationship between TP expression and measurements of hypoxia in human tumours. In this study, no relationship was demonstrated between TP expression and hypoxia. As previously discussed, stimuli other than hypoxia are involved in TP expression. TP is upregulated not only by cytokines (see Introduction), but also by low pH (Griffiths *et al*, 1997) and the activation of oncogenes such as c-erbB-2 and loss of Bcl-2 (Koukourakis *et al*, 1999). Another factor confounding the relationship between TP and hypoxia might relate to its role in stimulating angiogenesis. In comparison with wild-type cells, xenografted TP-overexpressing breast cancer cells were shown to be better oxygenated (measured using the comet assay). The authors suggested the finding was consistent with the TP-overexpressing tumours having an increased and functionally more competent vasculature (Griffiths and Stratford, 1998). Clearly, the relationships between hypoxia, angiogenesis and TP are unlikely to be simple. How the relationships might change during tumorigenesis, that is, from pre-malignant to early stage to advanced disease, remains to be explored.

Although TP is upregulated under hypoxia, we found no relationship between the expression of TP and HIF-1*α* in cervix tumours. As for TP, HIF-1*α* protein levels can be raised in tumours due to stimuli other than hypoxia: activation of oncogenes HER2 (Laughner *et al*, 2001), v-src (Jiang *et al*, 1997) and H-ras (Chen *et al*, 2001); loss of tumour suppressor gene function such as PTEN (Zundel *et al*, 2000) and signalling abnormalities such as MAPK (Richard *et al*, 1999). The relationship between TP and HIF has not been studied widely. While we found no relationship between TP and HIF-1*α* expression there was a borderline correlation with HIF-2*α*. This observation is consistent with a finding in human endometrial cancers of an association between tumour expression of TP and HIF-2*α* but not HIF-1*α* (Sivridis *et al*, 2002b). Recent evidence showed that whereas some genes are upregulated by HIF-1*α* and HIF-2*α*, others are preferentially activated by one or the other factor (Wang *et al*, 2005). It might be, therefore, that TP is preferentially induced by HIF-2*α*. Of interest is the observation that TP increased the hypoxic expression of HIF-1*α* in an orthoptic xenograft model of bladder cancer (Brown *et al*, 2005). This finding suggests that TP can be added to the long list of factors that promote HIF activation.

Consistent with the suggestion that TP promotes HIF-*α* expression, we found weak relationships between tumour TP expression and the expression of proteins upregulated by HIF (VEGF, CAIX and Glut-1). These results are also consistent with published evidence showing either a correlation or coexpression of TP with VEGF (Fujimoto *et al*, 1998; O'Byrne *et al*, 2000; van Triest *et al*, 2000) and CA-IX (Giatromanolaki *et al*, 2001). Studies have also shown that TP may stimulate VEGF expression. Increasing TP enzymatic activity induces oxidative stress which has been shown to upregulate various cytokines including VEGF (Brown *et al*, 2000). Furthermore, increased TP activity augments VEGF expression under hypoxic conditions *in vivo* (Brown *et al*, 2005). Our data support this relationship. TP and its enzymatic products have been shown to be angiogenic in various models (Moghaddam *et al*, 1995; Jones *et al*, 2002). These effects may be synergistic with or mediated by VEGF.

This study found TP expression to be a weak prognostic indicator in locally advanced carcinoma of the cervix. As we showed previously for VEGF (Loncaster *et al*, 2000), TP significantly predicted for metastasis-free survival but not local control. This is consistent with its role as a promoter of tumour angiogenesis rather than a role in hypoxia-associated radioresistance.

The exact role of TP in tumorigenesis and angiogenesis remains unclear. Studies have shown that the use of TP inhibitors in TP-overexpressing xenografted cell lines reduced angiogenesis and increased apoptosis (Matsushita *et al*, 1999). These results have not yet been developed into clinical trials. Nevertheless, owing to tumour-specific activating and deactivating mutations, the efficacy of TP inhibitors might vary not only between individual tumours but also between different types of tumours. Emphasis should be placed on elucidating the exact relationship of TP with other angiogenic factors, HIF, signalling abnormalities, oncogene activation and loss of tumour suppressor gene function. Such information should prove useful for the successful introduction of TP inhibition strategies into clinical practice.

## Figures and Tables

**Table 1:** Distribution of patients according to TP expression

		**TP score**				
**Parameter**	***N***	**0**	**1**	**2**	**3**	***P* a**
*Stage*						
I	32	6	10	11	5	0.13
II	34	2	9	9	14	
III	45	4	14	18	9	
IV	8	0	5	1	2	
*Age*						
<52 years	59	9	18	11	21	0.002
>52 years	60	3	20	28	9	
*Differentiation*						
Well	23	1	7	8	7	0.97
Moderate	74	8	24	24	18	
Poor	16	1	6	5	4	

a *P*=statistical significance using Fisher's exact test.

**Table 2:** Univariate log-rank analysis of putative prognostic factors for outcome following radiotherapy for carcinoma of the cervix

**Parameter**	***n***	**Disease-specific survival**	**Metastasis-free survival**	**Local control**
Stage	119	<0.001	0.005	0.11
Age	119	0.65	0.12	0.043
Differentiation	113	0.81	0.69	0.25
TP expression^a^	119	0.067	0.031	0.23

a Analysed using univariate Cox test for trend

The *P*-value for each factor is given.

**Table 3:** Bivariate stratified log-rank analyses showing the significance of the level of TP expression as a prognostic factor after allowing for the listed parameters

**Parameter**	***n***	**Disease-specific survival**	**Metastasis-free survival**	**Local control**
Stage	119	0.091	0.049	0.21
Age	119	0.19	0.053	0.25
Differentiation	113	0.21	0.078	0.30

The *P*-value for each factor is given.
